# Familial resemblances in human whole blood transcriptome

**DOI:** 10.1186/s12864-018-4698-6

**Published:** 2018-04-27

**Authors:** Bénédicte L. Tremblay, Frédéric Guénard, Benoît Lamarche, Louis Pérusse, Marie-Claude Vohl

**Affiliations:** 1 Institute of Nutrition and Functional Foods (INAF), Laval University, Pavillon des Services, 2440 Hochelaga Blvd, Quebec City, QC G1V 0A6 Canada; 2 CHU de Québec Research Center – Endocrinology and Nephrology, 2705 Laurier Blvd, Quebec City, QC G1V 4G2 Canada

**Keywords:** Gene expression, Familial resemblances, DNA methylation, Genetic correlations, Metabolic pathways

## Abstract

**Background:**

Considering the implication of gene expression in the susceptibility of chronic diseases and the familial clustering of chronic diseases, the study of familial resemblances in gene expression levels is then highly relevant. Few studies have considered the contribution of both genetic and common environmental effects to familial resemblances in whole blood gene expression levels. The objective is to quantify the contribution of genetic and common environmental effects in the familial resemblances of whole blood genome-wide gene expression levels. We also make comparisons with familial resemblances in blood leukocytes genome-wide DNA methylation levels in the same cohort in order to further investigate biological mechanisms.

**Results:**

Maximal heritability, genetic heritability, and common environmental effect were computed for all probes (20.6%, 15.6%, and 5.0% respectively) and for probes showing a significant familial effect (78.1%, 60.1%, and 18.0% respectively). Pairwise phenotypic correlations between gene expression and DNA methylation levels adjusted for blood cell heterogeneity were computed for probes showing significant familial effect. A total of 78 probe pairs among the 7,618,401 possible pairs passed Bonferroni correction (corrected *P*-value = 6.56 × 10^− 9^). Significant genetic correlations between gene expression and DNA methylation levels were found for 25 probe pairs (absolute genetic correlation of 0.97).

**Conclusions:**

Familial resemblances in gene expression levels were mainly attributable to genetic factors, but common environmental effect also played a role especially in probes showing a significant familial effect. Probes and CpG sites with familial effect seem to be under a strong shared genetic control.

**Electronic supplementary material:**

The online version of this article (10.1186/s12864-018-4698-6) contains supplementary material, which is available to authorized users.

## Background

Gene expression is increasingly studied in the context of chronic diseases due to the increasing evidence of its implication in disease susceptibility [1–3]. The nature of genetic variance for chronic diseases may result from hereditary changes impacting gene regulation rather than protein structure and function [3]. The study of the inheritance patterns and familial resemblances of gene expression is then highly relevant to better understand the origin of these diseases. Studies, including The Brisbane Systems Genetics Study (GSGS) have investigated the heritability of gene expression in whole blood [4–8]. However, few studies have considered the contribution of both genetic and common environmental effects to familial resemblances in gene expression levels [4, 6–8].

The relationship between gene expression and DNA methylation is well established [9]. Several studies have assessed the role of genetic factors on gene expression and DNA methylation [10–12]. Previous studies have also reported genetic heritability of DNA methylation levels ranging from 8 to 18% in whole blood [13–15]. A study by our group in French Canadians showed that familial resemblances in blood leukocytes genome-wide DNA methylation levels are mainly attributable to genetic factors, but that common environmental effect also plays a role [16]. A study by Shakhbazov et al. also demonstrated a strong, shared genetic control for DNA methylation and gene expression [17].

Our objective is thus to quantify the contribution of genetic and common environmental effects in familial resemblances of gene expression using whole blood genome-wide expression levels in a family-based sample of 48 French Canadians from 16 families. To further investigate biological mechanisms, we also compared results on gene expression with familial resemblances in blood leukocytes genome-wide DNA methylation levels in the same cohort.

## Results

### Correlations of gene expression levels between relative pairs

Mean absolute correlations across all 18,160 detected probes were calculated between pairs from normalized gene expression levels (Table 1). The mean correlation coefficients were 0.31 ± 0.21 among siblings (*n* = 13), 0.24 ± 0.17 for mother-offspring pairs (*n* = 26), 0.23 ± 0.16 for parent-offspring pairs (*n* = 37), and 0.021 ± 0.015 among unrelated individuals (*n* = 1078). Related individuals (siblings, mother-offspring, and parent-offspring pairs) had more similar genome-wide gene expression levels compared to unrelated individuals according to these correlations.

**Table 1:** Mean absolute correlations across all probes of normalised gene expression levels between relative pairs

Relative pairs	n	Correlation ± SD
Siblings	13	0.31 ± 0.21
Mother-Offspring	26	0.24 ± 0.17
Parent-Offspring	37	0.23 ± 0.16
Unrelated	1078	0.021 ± 0.015

*Abbreviation*: *SD* Standard deviation

### Gene expression heritability analyses

**Fig. 1:**
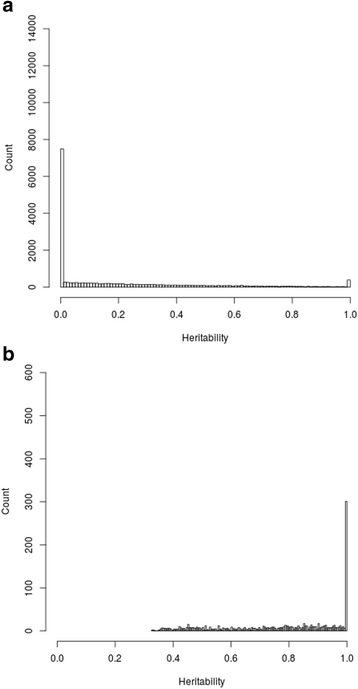
Distribution of maximal heritability estimates for gene expression levels of **a**) all probes (*n* = 18,160), **b**) significant probes (*n* = 1211)

Table 2 shows estimates of maximal heritability, genetic heritability, and common environmental effect from the full general model. When considering gene expression levels adjusted for blood cell heterogeneity of all probes (*n* = 18,160), we obtained a mean maximal heritability of 20.6%, a genetic heritability of 15.6%, and a common environmental effect of 5.0% (Table 2). We have previously quantified contribution of genetic and common environmental effects in familial resemblances in genome-wide DNA methylation levels, but analyses did not account for blood cell composition [16]. The analyses were reconducted taking into account the cell composition. We observed slightly lower values when computing maximal heritability, genetic heritability, and common environmental effect for all 472,494 cytosine-phosphate-guanine (CpG) sites (8.1%, 5.5%, and 2.6% respectively). DNA methylation heritability estimates adjusted for blood cell composition were similar but slightly lower than the ones previously reported, with maximal heritability of 12.7%, genetic heritability of 8.2%, and common environmental effect of 4.5% [16]. The distribution of maximal heritability estimates of gene expression levels of all probes (*n* = 18,160) with heritability ranging from 0 to 100% on the x-axis and the count (number of probes) on the y-axis is presented in Fig. 1a. A total of 7182 probes (39.5% of all probes) and 318 probes (1.8% of all probes) had an estimated maximal heritability of 0% and 100%, respectively.

**Table 2:** Heritability estimates of gene expression levels

Type of heritability estimates (% ± SD)	All probes (n = 18,160)	Significant probes (n = 1211)	FDR-corrected significant probes (n = 12)
Maximal heritability	20.62 ± 27.09	78.13 ± 20.70	94.63 ± 9.66
Genetic heritability	15.60 ± 26.49	60.11 ± 37.90	50.87 ± 23.77
Common env. effect	5.02 ± 10.07	18.01 ± 19.59	43.75 ± 14.50

*Abbreviation*: *env* environmental, *SD* Standard deviation

**Fig. 2:**
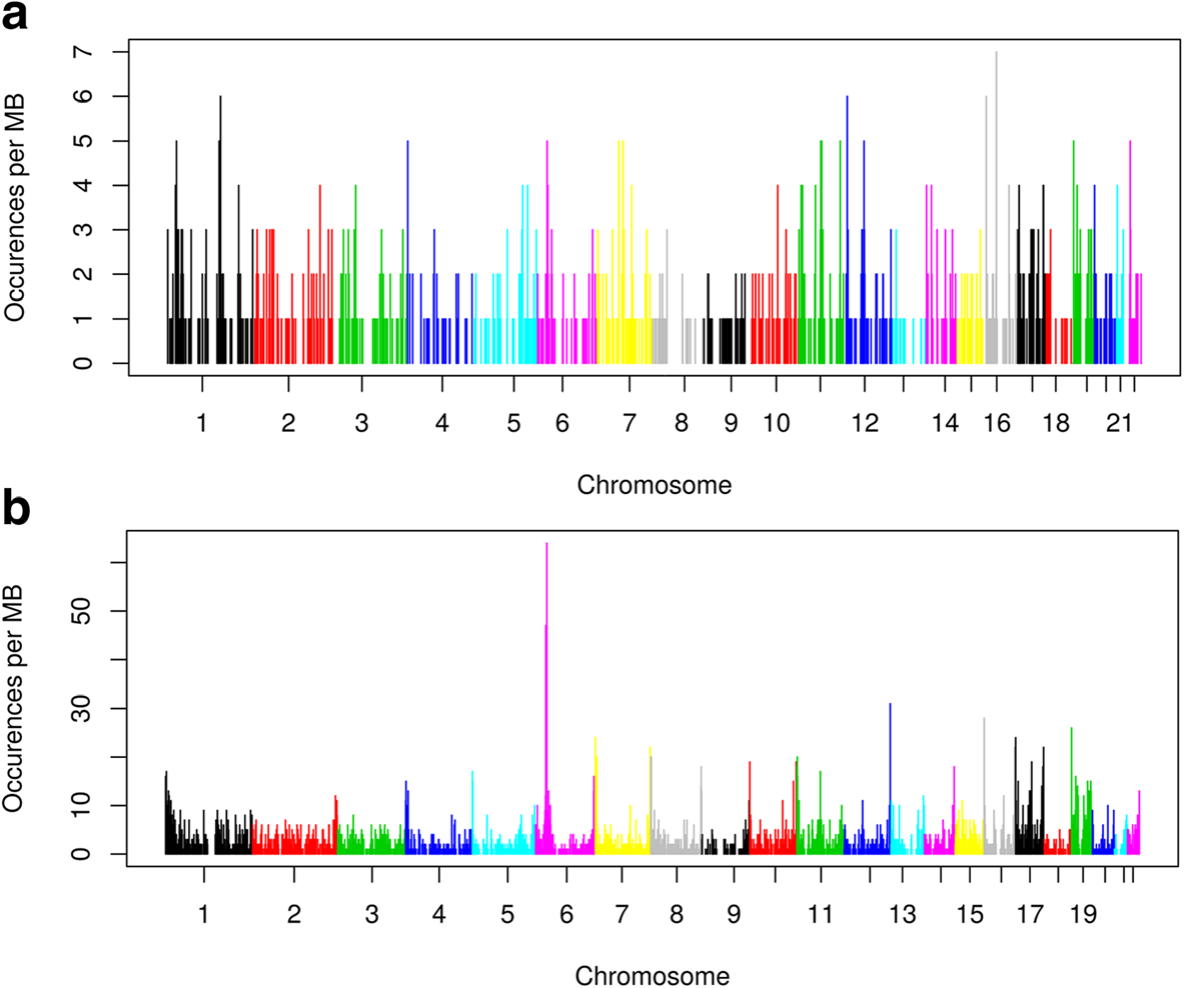
Chromosomal representation of annotated **a**) probes and **b**) CpG sites showing significant familial effect in gene expression and DNA methylation levels, respectively. Legend: All positions are from the Genome Build 37

**Fig. 3:**
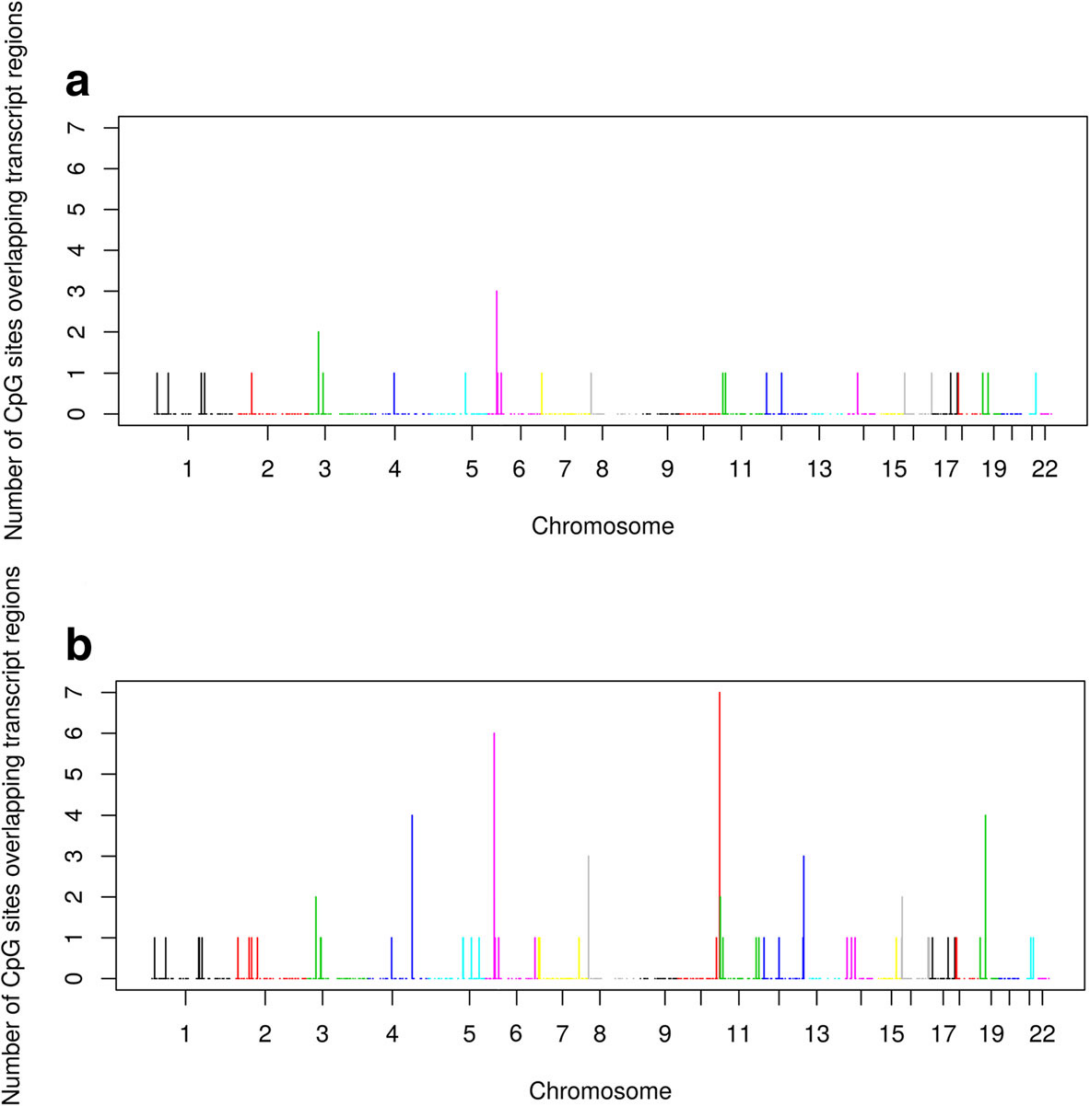
Chromosomal representation of number of significant CpG sites overlapping significant transcript regions **A)** ± 2 kb and **B)** ± 5 kb. Legend: All positions are from de Genome Build 37

Moreover, when considering the 1211 probes showing a significant (*P* ≤ 0.05) familial effect, a mean maximal heritability of 78.1%, a genetic heritability of 60.1%, and a common environmental effect of 18.0% were observed (Table 2). We observed similar estimates in DNA methylation analysis when computing maximal heritability, genetic heritability, and common environmental effect for the 6291 CpG sites showing a significant familial effect (66.4%, 39.6%, and 26.8% respectively). Once again, methylation heritability estimates adjusted for blood cell heterogeneity were very similar to the ones previously reported, with maximal heritability of 63.9%, genetic heritability of 39.3%, and common environmental effect of 24.6% [16]. When maximal heritability estimates of significant probes are plotted, the distribution shifts to the right, in accordance with the higher maximal heritability estimates ranging from 32.8 to 100% (Fig. 1b). A total of 255 probes (21.1% of all significant probes) gave an estimated maximal heritability of 100% (genetic heritability ranges from 0 to 100% and common environmental effect ranges from 0 to 72.1%). Distributions of genetic heritability and common environmental effect for all probes (*n* = 18,160) and significant probes (*n* = 1211) are illustrated in Additional files 1 and 2. Chromosomal representation of probes (n = 1211) and CpG sites (*n* = 6291) showing significant familial effect on gene expression and DNA methylation levels respectively are presented in Fig. 2. Heritable probes and CpG sites are distributed across the genome. The visible peak on chromosome 6 corresponds to the Major Histocompatibility Complex (MHC) [18]. Figure 3 depicted the number of significant CpG sites overlapping significant transcript regions (± 2 kb and ± 5 kb). CpG sites and probes showing significant familial effect were not very closely located.

Pairwise phenotypic correlations were computed for gene expression levels of the 1211 probes and DNA methylation levels of the 6291 CpG sites showing significant familial effect. Gene expression and DNA methylation levels adjusted for blood cell heterogeneity were significantly correlated in 78 probe pairs among the 7,618,401 possible pairwise tests after Bonferroni correction (corrected *P* = 0.05/ (1211 × 6291) = 6.56 × 10^− 9^) (Additional file 3). Further, genetic correlations between gene expression and DNA methylation levels for all 78 probe pairs passing Bonferroni threshold were estimated and genetic correlations were obtained for 39 probe pairs. Genetic correlations estimated the additive genetic effect that is shared between gene expression and DNA methylation levels. A total of 25 probe pairs had a significant genetic correlation (*P* ≤ 0.05) and the mean absolute value of genetic correlation was 0.97 (Additional file 4). Only two probe pairs had a genetic correlation passing Bonferroni correction (corrected *P* = 0.05/ 39 = 0.00128) (Table 3). The first probe pair was composed of cg22561794 located within the gene *BTNL8* on chromosome 5 and transcript ID_3520128 (NM_016437) located on chromosome 17. The second probe pair comprised cg02797144 located in an intergenic region on chromosome 16 and transcript ID_3990435 (BX282075) on chromosome 7.

**Table 3:** Top two probe pairs showing a significant genetic correlation

Transcript	CpG site	h2 expression ± SE	h2 methylation ± SE	Correlation coefficient	*P*-value
ID_3520128 (NM_016437, *TUBG2*, Chr17)	cg22561794 (*BTNL8*, Chr5)	0.63 ± 0.22	0.71 ± 0.20	−0.96	0.00116
ID_3990435 (BX282075, *HS.511718*, Chr7)	cg02797144 (Intergenic, Chr16)	0.64 ± 0.22	0.65 ± 0.19	− 1	0.00118

*Abbreviations*: *Chr* Chromosome, *h2* Heritability, *SE* Standard error. All positions are from de Genome Build 37

Lastly, heritability analysis was repeated for probes showing a significant familial effect after False Discovery Rate (FDR) correction (*P* ≤ 0.05). We obtained 12 FDR-corrected significant probes, assigned to 12 genes (*SPAG7*, *TMEM141*, *ZCCHC11, NDUFA2*, *CBL*, *HNRNPM*, *OXT*, *POLE4*, *MGC4677*, *DYNLT1*, *SOS1*, and *TBCA*), giving a mean maximal heritability of 94.6% (range 72.1 to 100%), a genetic heritability of 50.9% (range 0 to 74.1%), and a common environmental effect of 43.8% (ranges 25.9 to 72.1%) (Table 4). Six probes (*SPAG7, NDUFA2, CBL, HNRNPM, POLE4, MGC4677*) had an estimated maximal heritability of 100%. No CpG site in DNA methylation analysis passed the Bonferonni correction. Without the adjustment for blood cell heterogeneity, a total of 105 FDR-corrected significant CpG sites showed a mean maximal heritability of 81.8%, a genetic heritability of 26.9%, and a common environmental effect of 54.9% [16].

**Table 4:** Heritability estimates of false discovery rate-corrected significant probes

Transcript	Maximal heritability	Genetic heritability	Common env. effect	*P* (corrected *P*)*
ID_3440070 (NM_004890, *SPAG7*, Chr17)	100.0	57.86	42.14	3 × 10^− 6^ (0.029)
ID_5130113 (NM_032928, *TMEM141*, Chr9)	83.64	26.04	57.60	4 × 10^− 6^ (0.029)
ID_4260093 (NM_001009882, ZCCHC11, Chr1)	97.74	52.07	45.67	6 × 10^− 6^ (0.029)
ID_6840189 (NM_002488, *NDUFA2*, Chr5)	100.0	74.11	25.89	8 × 10^− 6^ (0.029)
ID_6960209 (NM_005188, *CBL*, Chr11)	100.0	66.75	33.25	8 × 10^− 6^ (0.029)
ID_2360669 (NM_031203, *HNRNPM*, Chr19)	100.0	58.45	41.55	1 × 10^− 5^ (0.030)
ID_1850372 (NM_000915, *OXT*, Chr20)	72.08	0	72.08	2 × 10^− 5^ (0.036)
ID_2120286 (NM_019896, *POLE4*, Chr2)	100.0	67.63	32.37	2 × 10^− 5^ (0.036)
ID_6350189 (NM_052871, *MGC4677*, Chr2)	100.0	64.83	35.17	2 × 10^− 5^ (0.036)
ID_6350634 (NM_006519, *DYNLT1*, Chr6)	100.0	67.11	32.89	2 × 10^− 5^ (0.036)
ID_2140519 (NM_005633, *SOS1*, Chr2)	82.07	15.11	66.96	3 × 10^− 5^ (0.045)
ID_5960093 (NM_004607, *TBCA*, Chr5)	100.0	60.54	39.46	3 × 10^−5^ (0.045)

*Abbreviations*: *Chr* Chromosome, *env* environmental, *FDR* False discovery rate. *P*-value of familial effect (Vg and Vc significantly different from 0). * False discovery rate-corrected *P* values. All positions are from de Genome Build 37

**Fig. 4:**
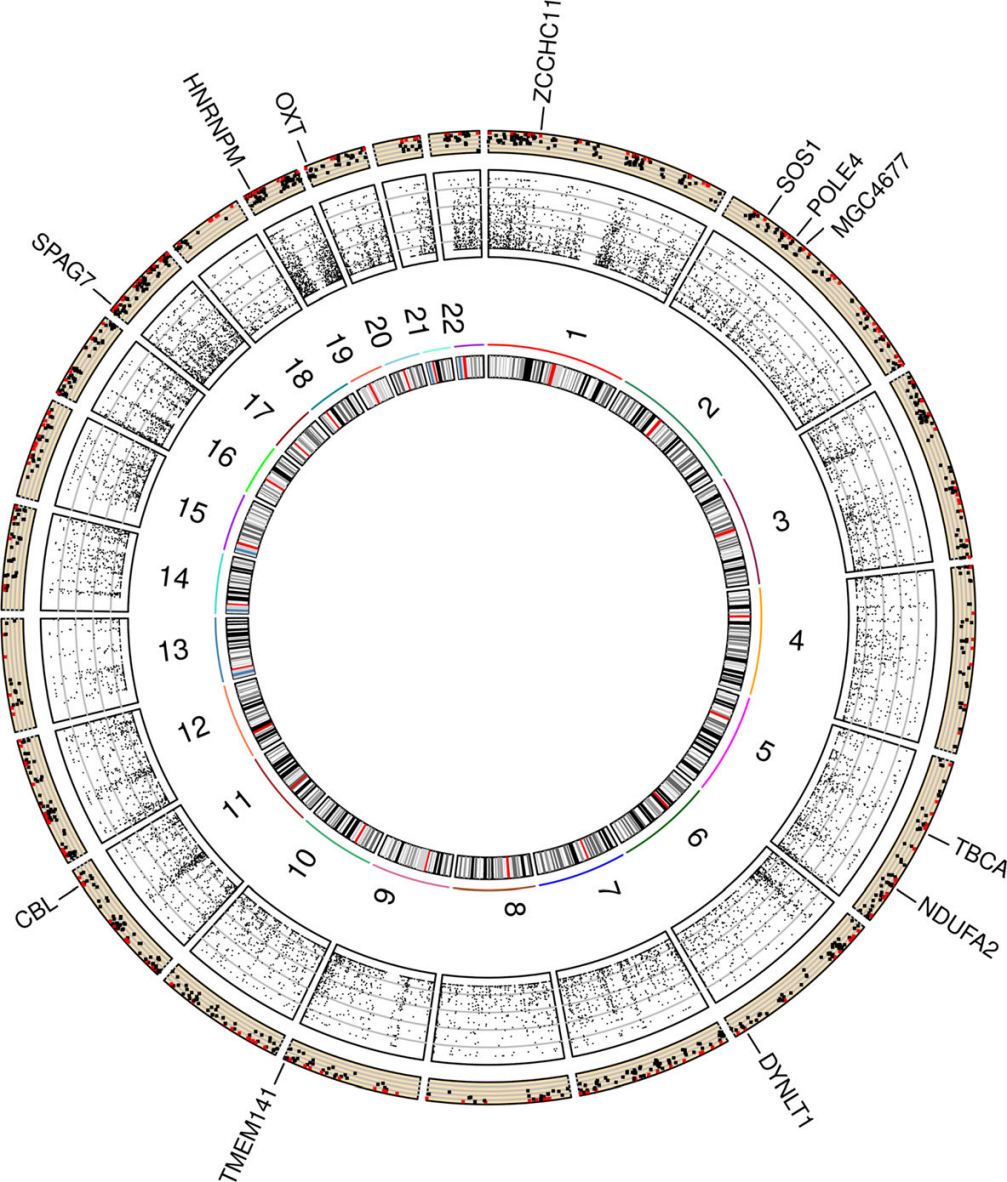
Circos plot depicting the distribution of maximal heritability estimates of gene expression across the genome. Legend: Moving from inner to outer circles, first circle represents chromosomes. Maximal heritability of all 18,160 probes has been represented in second circle as scatter plot (values ranging from 0 to 100%). Third circle represents maximal heritability of the 1211 probes showing a significant familial effect as scatter plot. Genes name of the 12 probes that passed FDR correction are also represented

Moreover, Fig. 4 shows Circos plot depicting the distribution of maximal heritability estimates of gene expression across the genome. These 12 FDR-corrected significant probes were assigned to 12 genes as illustrated in Fig. 3. There is also a visible higher maximal heritability in the MHC region on chromosome 6. A total of 14 probes out of 1211 were located in the MHC region, and were associated with the *HLA-F, HLA-H, HLA-A, HCG2P7, PPP1R11, HCP5, LST1, BAT2, C6ORF25, C6ORF27, LOC401252, HLA-DRB1, PSMB9, TAP2* genes.

### Pathways analyses

Ingenuity Pathway Analysis (IPA) revealed that 140 pathways were significantly (*P* ≤ 0.05) overrepresented among genes of the 1211 probes with a significant familial effect (Additional file 5). These pathways were related to inflammatory and immune response (*n* = 52), cell cycle regulation (*n* = 35), cancer (*n* = 17), DNA and RNA regulation (*n* = 11), intracellular and second messenger signaling (*n* = 10), cardiovascular signaling (*n* = 7), disease specific pathways (n = 5), and nuclear receptor signaling (n = 3). Interestingly, 22 out of the 140 pathways were in common with the 75 significantly overrepresented pathways among genes of the 6291 CpG sites with significant familial effect (Additional file 6). The detailed list of those 22 common pathways is presented in Additional file 7.

## Discussion

The first aim of this study was to quantify the contribution of genetic and common environmental effects in familial resemblances of gene expression levels in a family-based sample of 48 French Canadians from 16 families. Mean absolute correlations between relative pairs suggest an underlying genetic similarity. Similar absolute correlations for DNA methylation levels were observed in the same cohort [16]. In accordance with other studies, genetic factors seem to be the major determinant of familial resemblances in gene expression levels [4, 5, 17, 19]. Indeed for all probes (*n* = 18,160), significant probes (*n* = 1211), and FDR-corrected significant probes (n = 12), familial effect was mainly due to genetic heritability. FDR-corrected significant probes were assigned to 12 genes (*SPAG7*, *TMEM141*, *ZCCHC11, NDUFA2*, *CBL*, *HNRNPM*, *OXT*, *POLE4*, *MGC4677*, *DYNLT1*, *SOS1*, and *TBCA*).

Using an alternative genetic model for comparison purposes, we obtained a genetic heritability estimate for all probes of 22.8%, which is higher than estimate reported in Huan et al. [5]. Using the same alternative model, a total of 7682 probes (42.3% of all probes) had a genetic heritability > 0 compared to 14,753 probes (82% of all probes) in Powell et al. [4]. In the same manner, we obtained 2097 probes with a genetic heritability > 0 (*P* ≤ 0.05) compared to 7161 genes in Huan et al. [5]. Overall, we reported higher genetic heritability estimates but lower number of probes with genetic heritability > 0. This may be explained by the small sample size of the present study that limits the statistical power to detect low heritability estimates. In fact, in a sample of 648 twins, Grundberg et al. argued that this number was not sufficient to obtain reliable heritability estimates of less than 10% [8, 20]. Thus, the small sample size upon which the present study is based on may affect the reliability of low values of heritability estimates and partly explain the higher overall genetic heritability estimate and lower number of probes with genetic heritability > 0 compared to other studies.

Moreover, a total of 6555 probes had a common environmental effect > 0 in the full general model compared to 3373 probes in Powell et al. [4]. These results should however be compared with caution considering that Powell et al. used a different general model which includes additive, non-additive and common environmental effects and did not account for blood cell heterogeneity [4]. Grundberg et al. also reported a common environmental component in 32% of expressed lymphoblastoid cell line transcripts [8]. Interestingly, Nédélec et al. demonstrated that expression of several genes in macrophages is different between subjects of European and African ancestry [21]. All subjects in the present study are French Canadian Caucasians of French Canadian descent living in the same city (Quebec City). Thus we can hypothesize that gene expression levels is more homogeneous in a founder population of European descent [22].

The secondary aim was to compare results of the present study with results on familial resemblances in genome-wide DNA methylation levels using the same cohort in order to further investigate biological mechanisms. We first reported that familial resemblances in genome-wide gene expression and DNA methylation levels are mainly due to genetic effects with a contribution of common environmental effect. CpG sites and probes with a strong familial effect were distributed across the genome and were not very closely located (± 2 and 5 kb). This is in accordance with a study by Van Eijk KR et al. that found that expression and methylation modules (clusters of interconnected genes) exhibit relatively few overlapping genes, although some of the overlaps were statistically significant [12]. Grundberg et al. also showed that over 60% of gene expression heritability is *trans* to the structural gene [8]. Shakhbazov et al. demonstrated that gene expression and DNA methylation probe pairs with shared QTL(s) have larger genetic correlations in contrast with the same chromosome probe pairs without shared QTL [17]. A study by Price et al. using 722 Icelanders from family cohorts demonstrated that the proportion of gene expression heritability attributable to *cis* regulation was 37% in blood [23]. The proportion of heritability of gene expression attributable to *cis* regulation is also expected to increase as a function of the number of different cell types [23]. We could therefore hypothesise that the adjustment for blood cell composition attenuated the proportion of heritability attributable to *cis* regulation.

Regarding the 78 significant phenotypic correlations between DNA methylation and gene expression levels, only three probe pairs were located on the same chromosome. This suggests that *cis* regulation of single nucleotide polymorphisms (SNPs) may not be responsible for genetic heritability of probes and phenotypic correlations with methylation levels of CpG sites. We reported 25 significant genetic correlations between gene expression and DNA methylation levels adjusted for blood cell heterogeneity, thus suggesting a shared genetic control. We reported higher genetic correlation (− 0.97/ 0.97, for negative and positive genetic correlations, respectively) than Shakhbazov et al. (− 0.69/0.68) [17]. This could be explained by the fact that they calculated correlations between gene expression and DNA methylation of probe pairs across the genome (5 × 10^9^), while we restrained analyses to probes and CpG sites with a significant familial effect (7 × 10^6^). Shakhbazov et al. also demonstrated that correction for cell heterogeneity greatly impacts correlations between genome-wide DNA methylation and gene expression levels with a 300 times reduction in number of probe pairs passing Bonferonni correction. Accordingly, we also observed a 48 times reduction (from 1211 to 25 significant genetic correlations) after the correction for blood cell composition in our subset of significant probes and CpG sites. A total of two out of 25 probe pair correlations remained significant after Bonferroni correction. The first probe pair comprised cg22561794 on the *BTNL8* gene encoding for butyrophilin like 8 and NM_016437 on *TUBG2* gene encoding for the tubulin gamma 2. BTNL8 gene is involved in immune response as it stimulates cytokine production and is also altered in intestinal inflammation and colon cancer [24, 25]. TUBG2 gene is primarily detected in the brain and its expression seems to be closely related to oncogenesis [26, 27]. The second probe pair comprised cg02797144 located in an intergenic region on chromosome 16 and BX282075 expression probe measuring *HS.511718* on chromosome 7.

We also observed similarities in overrepresented pathways of significant probes and CpG sites. Indeed, 22 pathways were in common between overrepresented pathways (*n* = 140) of significant probes and significant CpG sites (*n* = 75) [16]. Thus, some CpG sites and probes with a familial effect seem to be implicated in the same metabolic pathways. The majority of these 22 common pathways were related to inflammatory and immune response, which is in line with the 14 highly heritable probes (mean maximal heritability of 82.0%) found in the MHC region on chromosome 6 [18]. Highly heritable CpG sites in the MHC region were also reported by us [16] and others [13]. This result is also in line with the reported ancestry-associated differences in the gene regulatory response to infection [21].

This study has its own strengths and limitations. The main strength is the comparison of results on familial resemblances in gene expression with results on familial resemblances in DNA methylation levels in the same cohort. The use of a cell type predictor both in methylation and expression analysis allow to greatly attenuate the bias associated with blood cell heterogeneity, especially in phenotypic and genetic correlations. The calculation of genetic correlations adds important information about the additive genetic effect that is shared between gene expression and DNA methylation levels. To the best of our knowledge this is the first study on familial resemblances in genome-wide gene expression levels carried out on healthy French Canadians, a founder population of European descent. Regarding statistical analysis, a filter was applied to exclude lowly expressed genes and thus avoiding bias in estimates of gene expression heritability. Yang et al. reported that genes with very low expression levels have a reduced statistical power to be detected for a significant genetic component [28]. Adjustments were also made to account for multiple testing.

Regarding limitations of the study, the main one is the small sample size which has been discussed earlier. The fact that we do not have genotypes for study subjects represents another limitation considering the impact of SNPs on gene expression levels. Indeed, an eQTL analysis using 862 subjects from the GSGS revealed 15,000 associations of SNPs with gene expression levels [3]. We also used a cell type predictor in methylation and expression analysis to reduce the effect of blood cell heterogeneity. However, Shakhbazov et al. demonstrated that correction for predicted cell counts is not sufficient to remove the effect of blood cell heterogeneity on the correlations between DNA methylation and gene expression levels [17]. Using the observed cell proportions remains the best way to remove this effect as much as possible [17]. Moreover, certain methodological considerations in pathway analysis including the fact that annotation of genetic variants is inconsistent across databases, incomplete and biased toward known genes must be noted [29]. The gold standard to establish validity of findings from pathway analysis remains the replication of results in independent studies.

## Conclusion

In conclusion, familial resemblances in gene expression levels are mainly attributable to genetic factors but common environmental effect also plays a role especially in probes showing a significant familial effect. Heritability estimates of genome-wide gene expression are similar but higher than those of genome-wide DNA methylation. Finally, pairwise phenotypic correlations between gene expression and DNA methylation of probes with familial effect are mainly attributable to a shared genetic control as showed by high genetic correlations.

## Methods

### Patients and design

A total of 48 subjects from 16 families were recruited in the Greater Quebec City metropolitan area, in Canada, as part of the GENERATION Study. Families were composed of 16 mothers, 6 fathers, and 26 children. To be eligible parents had to be the biological parents of their child (or children). Parents had to be in good general health, non-smokers, with body mass index ranging between 18 and 35 kg/m^2^, and free of any metabolic conditions requiring treatment although the use of Synthroid® (levothyroxine) or oral contraceptive was tolerated. Families living under the same roof comprised at least the mother and one child aged between 8 and 18. Children also had to be non-smokers and in good general health. They were not eligible if using psycho-stimulators [Ritalin® (methylphenidate), Concerta® (methylphenidate), and Strattera® (atomoxetine)]. Parents and children were asked to complete several dietary, physical activity, medical history, and pregnancy questionnaires under the supervision of a registered dietitian during their visit at the Institute of Nutrition and Functional Foods (INAF). The experimental protocol was approved by the Ethics Committees of Laval University Hospital Research Center and Laval University. All participants (adults and children) signed an informed consent document. Parental consent was also obtained by signing the child consent document.

### Anthropometric and metabolic measurements

Body weight, waist girth, and height were measured according to the procedures recommended by the Airlie Conference. [30] Blood samples were collected from an antecubital vein into PAXgene™ tubes after 12-h overnight fast and 48-h alcohol abstinence.

### RNA extraction and gene expression analysis

Total RNA was isolated and purified from whole blood using PAXgene Blood RNA Kit (QIAGEN). Quantification and verification of the purified RNA was assessed using both the NanoDrop (Thermo Scientific, Wilmington, DE, USA) and the 2100 Bioanalyzer (Agilent Technologies, Cedar Creek, TX, USA). The HumanHT-12 v4 Expression BeadChip (Illumina Inc., San Diego, CA) was used to measure expression levels of ~ 47,000 probes (> 31,000 annotated genes). This was performed at the McGill University and Genome Quebec Innovation Centre (Montreal, Canada). The FlexArray software (version 1.6) [31] and the lumi R package were used to analyze and normalize gene expression levels. More specifically, robust multi-array analysis, variance stabilizing transformation, and quantile normalization were used for background correction, variance stabilization, and normalization, respectively. Probes with a detection *P*-value ≤0.05 in at least 25% of all subjects were considered in analysis. A total of 18,160 probes among the 47,323 probes on the microarray (38.4%) showed significant gene expression in blood and were used for heritability analysis. This results is similar to the one reported in the BSGS (17,926 probes with a detection *P*-value ≤0.05 in 10% of all 862 subjects) [3]. Details about DNA extraction and genome-wide DNA methylation analysis of the autosomal 472,494 CpG sites detected in more than 90% of all subjects (*P*-value > 0.01) and mapped to unique location are presented in a previous paper on the same cohort [16].

### Adjustment for blood cell heterogeneity

In methylation analysis, the cell proportions were predicted with the Jaffe et al. algorithm for all 48 subjects [32]. This algorithm has been chosen because it was adjusted for the Illumina HumanMethylation450k [32]. We obtained estimated cell counts for six different cell types: CD8^+^ T cells, CD4^+^ T cells, NK cells, B-cells, monocytes, and granulocytes. In expression analysis, the cell proportions were predicted with the Abbas et al. algorithm for all subjects [33]. This algorithm has been chosen because it was developed specifically for blood [33]. We obtained estimated cell counts for 17 different cell types (resting helper T cells, activated helper T cells, resting cytotoxic T cells, activated cytotoxic T cells, resting B cells, activated B cells, BCR-ligated B cells, IgM memory B cells, IgA/IgG memory B cells, plasma cells, resting NK cells, activated NK cells, monocytes, activated monocytes, resting dendritic cells, activated dendritic cells, and neutrophils) [33] that have been grouped in three classes: lymphocytes, neutrophils, and monocytes.

### Statistical analysis

R software v2.14.1 (R Foundation for Statistical Computing; http://www.r-project.org) [34] was used to compute the mean absolute correlation of raw gene expression levels between relative pairs across all 18,160 probes. For heritability analysis of gene expression and DNA methylation levels, corrections were made for the effects of microarray, position on microarray, sex, age, age^2^, sex*age, sex*age^2^, and blood cell counts (estimated cell counts for six cell types in methylation analyses and three cell types in gene expression analyses), using a standard least squares model in JMP software v12. We used residuals from this model to compute heritability estimates using the variance component method implemented in QTDT v2.6.1. [35] We used full general models in which the variance in gene expression levels of each probe and DNA methylation levels of each CpG site were partitioned into polygenic effects (Vg), common environmental effects shared by family members (Vc), and non-shared environmental effects unique to each individual (Ve). We tested this full general model against a null model of no familial resemblance in which Vg = Vc = 0. We then computed mean maximal heritability, as the proportion of variance accounted by genetic and common environmental effects (Vg + Vc/Vg + Vc + Ve), mean genetic heritability, as the proportion of variance accounted by genetics effects (Vg/Vg + Vc + Ve), and the mean common environmental effect as the proportion of variance accounted by common environmental effects (Vc/Vg + Vc + Ve). Additionally, we computed heritability estimates in probes and CpG sites showing a significant familial effect (Vg and Vc significantly different from zero (*P* ≤ 0.05)). We computed FDR-corrected *P*-values to account for multiple testing. For comparison purposes, we computed alternative genetic models in which the variance in gene expression and DNA methylation levels was partitioned into Vg and Ve. We then computed mean genetic heritability, as the proportion of variance accounted by genetic effects (Vg/Vg + Ve).

Phenotypic Pearson correlations between DNA methylation and gene expression levels were computed using R software v2.14.1 [34]. Assuming bivariate normal distribution, asymptomatic *P*-values were computed based on Fisher Z transformation as in Shakhbazov et al. for comparison purposes [17]. Genetic correlations between DNA methylation and gene expression were calculated using a bivariate analysis in SOLAR Eclipse version 7.6.4. Bonferonni corrections were used to account for multiple testing in phenotypic and genetic correlations. We used RCircos package and a modified version of the qqman package in R software to make the Circos plot and the chromosomic representations. [36]. Finally, we used the IPA system (Ingenuity® Systems, www.ingenuity.com) to analyze pathways overrepresented among genes of significant probes (*n* = 1211).

## Additional files

Additional file 1: Distribution of genetic heritability estimates for gene expression levels of A) all probes (*n* = 18,160), B) significant probes (*n* = 1211). Histogram of genetic heritability estimates for all and significant probes. (TIF 491 kb)

Additional file 2: Distribution of common environmental effect estimates for gene expression levels of A) all probes (n = 18,160), B) significant probes (*n* = 1211). Histogram of common environmental effect estimates for all and significant probes. (TIF 481 kb)

Additional file 3: Significant phenotypic correlations between DNA methylation and gene expression levels (*n* = 78). List of all 78 probe pairs that showed a significant (*P* ≤ 0.05) phenotypic correlation between DNA methylation and gene expression levels. (XLSX 19 kb)

Additional file 4: Probe pairs showing a significant genetic correlation (*n* = 25). List of all 25 probe pairs that showed a significant (*P* ≤ 0.05) genetic correlation between gene expression and DNA methylation levels. (XLSX 10 kb)

Additional file 5: Overrepresented pathways identified among genes of probes with a familial effect (n = 1211). Table describing all 140 significant overrepresented pathways identified from gene expression analysis (IPA canonical pathways, associated *P*-value, and list of differentially expressed genes). (XLSX 18 kb)

Additional file 6: Overrepresented pathways identified among genes of CpG with a familial effect (*n* = 6291). Table describing all 75 significant overrepresented pathways identified from DNA methylation analysis (IPA canonical pathways, associated *P*-value, and list of differentially methylated genes). (XLSX 17 kb)

Additional file 7: Common overrepresented pathways identified among genes with significant familial effect in gene expression levels (n = 1211) and DNA methylation (n = 6291). Table describing all 22 significant overrepresented pathways in common between genes with significant familial effect in gene expression and DNA methylation levels (IPA canonical pathways and list of differentially expressed genes). (XLSX 10 kb)

## Abbreviations

CpG: Cytosine-phosphate-guanine
eQTL: Expression quantitative trait loci
FDR: False discovery rate
GSGS: The Brisbane Systems Genetics Study
INAF: Institute of Nutrition and Functional Foods
IPA: Ingenuity Pathway Analysis
MHC: Major Histocompatibility Complex
SNPs: Single nucleotide polymorphisms
