# Supplementary figures and images for: Familial resemblances in human whole blood transcriptome
Source: BMC Genomics. 2018 Apr 27;19:300. doi: 10.1186/s12864-018-4698-6 (PMC5921553; doi:10.1186/s12864-018-4698-6)

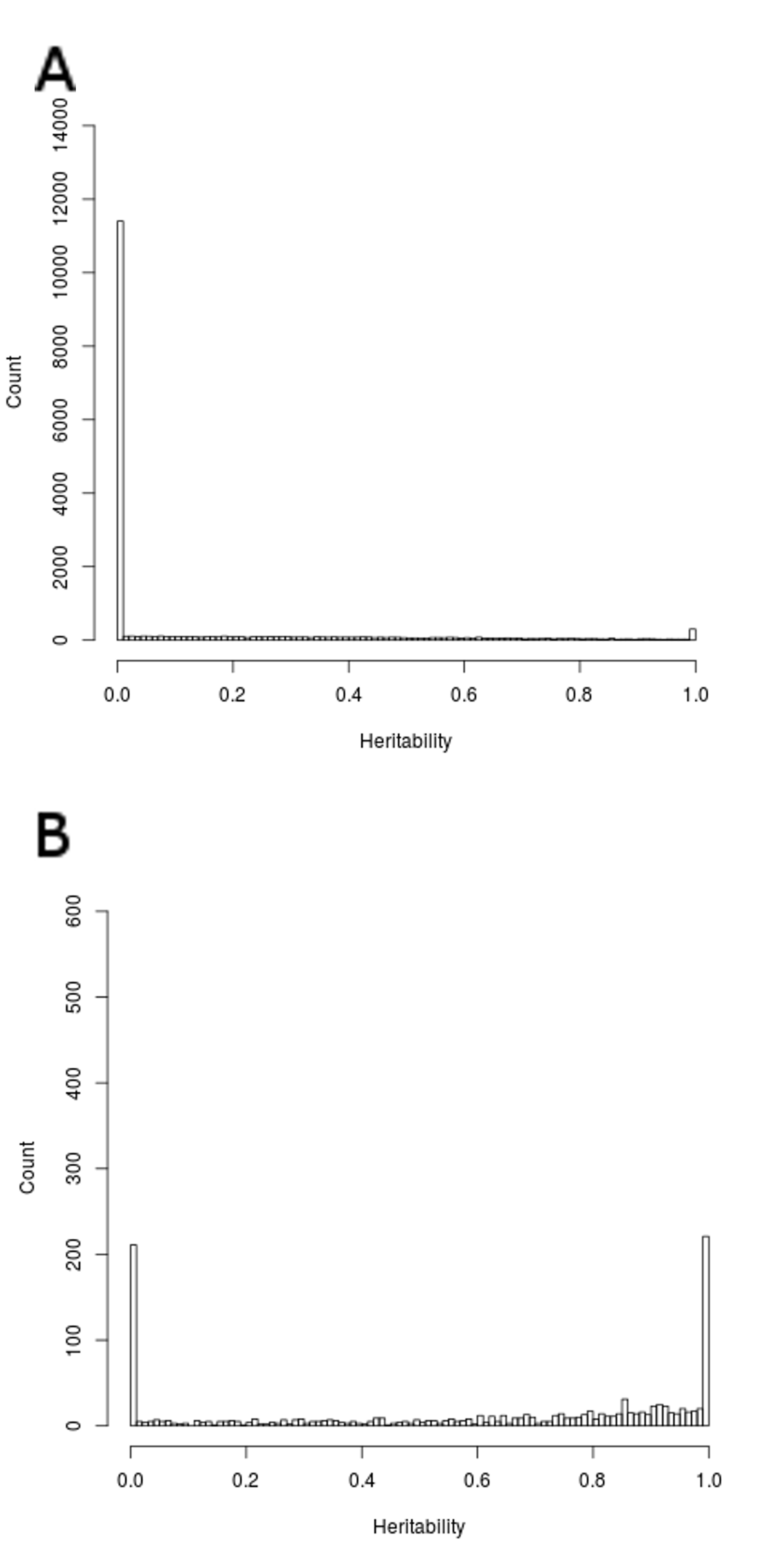

Supplement: Supplementary file 1 — Distribution of genetic heritability estimates for gene expression levels of A) all probes (n = 18,160), B) significant probes (n = 1211). Histogram of genetic heritability estimates for all and significant probes. (TIF 491 kb) [file 12864_2018_4698_MOESM1_ESM.tif]

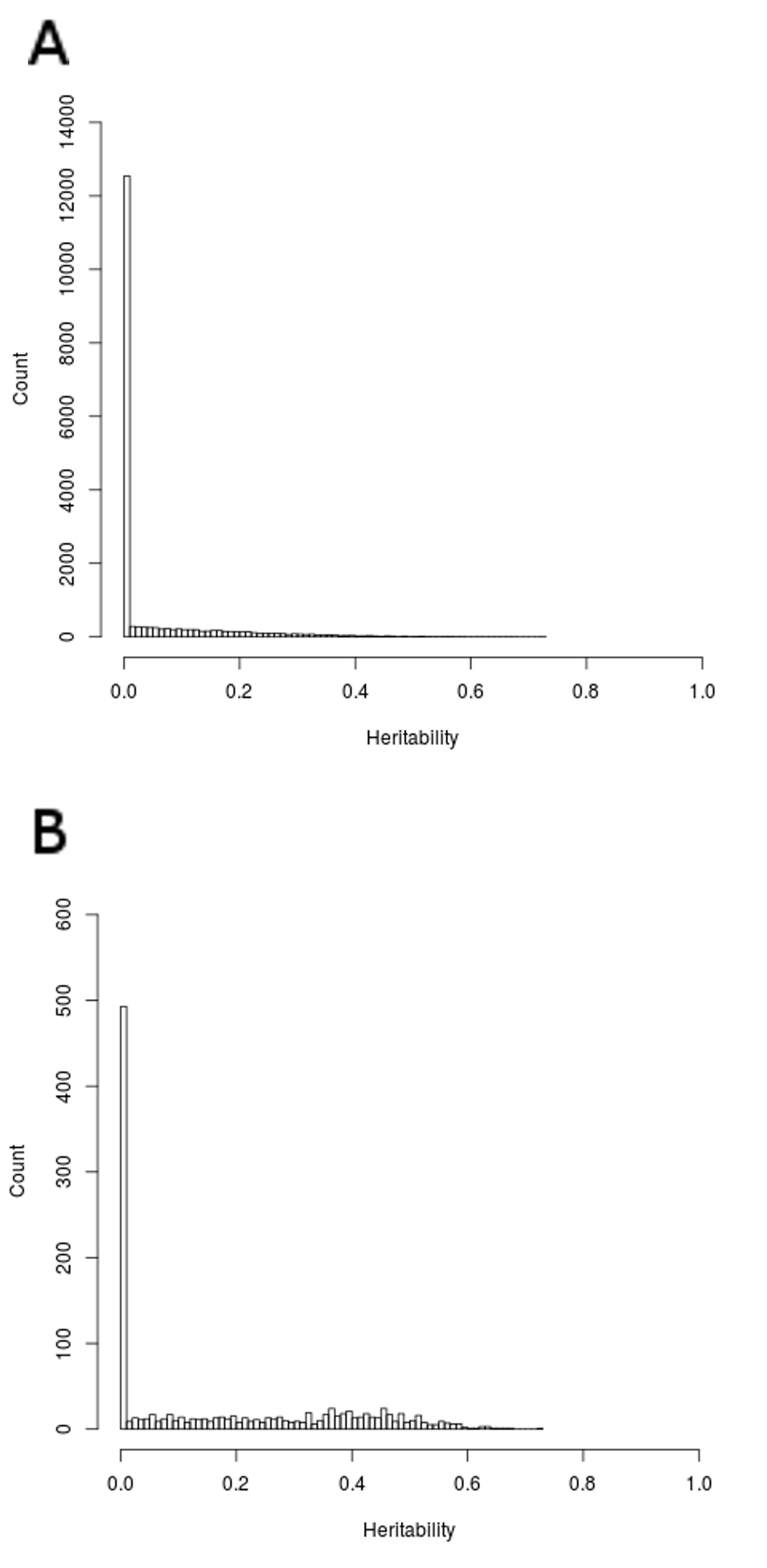

Supplement: Supplementary file 2 — Distribution of common environmental effect estimates for gene expression levels of A) all probes (n = 18,160), B) significant probes (n = 1211). Histogram of common environmental effect estimates for all and significant probes. (TIF 481 kb) [file 12864_2018_4698_MOESM2_ESM.tif]
